# Phase Evolution of High-Entropy Stannate Pyrochlore Oxide Synthesized via Glycine-Assisted Sol–Gel Synthesis as a Thermal Barrier Coating Material

**DOI:** 10.3390/nano15120939

**Published:** 2025-06-17

**Authors:** Mariappan Anandkumar, Kannan Pidugu Kesavan, Shanmugavel Sudarsan, Dmitry Evgenievich Zhivulin, Natalia Aleksandrovna Shaburova, Ahmad Ostovari Moghaddam, Ksenia Sergeevna Litvinyuk, Evgeny Alekseevich Trofimov

**Affiliations:** 1 High-Entropy Materials Research Laboratory, South Ural State University, Chelyabinsk 454080, Russia; 2 Department of Physics, PSG Institute of Technology and Applied Research, Coimbatore 641 062, India; 3 Department of Chemistry, Saveetha Engineering College, Chennai 602 105, India; 4 Laboratory of Problems of Recycling Modern Multicomponent Materials with Complex Structure, South Ural State University, Chelyabinsk 454080, Russia; 5 Regional Youth Laboratory of Electromechanical, Electronic and Electrochemical Systems, South Ural State University, Chelyabinsk 454080, Russia; 6 Department of Materials Science, Physical and Chemical Properties of Materials, South Ural State University, Chelyabinsk 454080, Russia; 7 Department of Applied Mathematics, National Research University Higher School of Economics, Moscow 101000, Russia

**Keywords:** high-entropy pyrochlore oxide, glycine-assisted sol–gel, phase evolution, thermal expansion coefficient, hardness

## Abstract

High-entropy ceramics have gained wider attention due to their structural integrity and stability, which can be used in various functional applications. Especially, high-entropy oxides exhibit excellent thermal stability, particularly at high temperatures. Thermal barrier coating materials must demonstrate good thermal stability without any phase transformation or phase separation, which is critical in aerospace and energy conversion applications. To address this, we have prepared new high-entropy stannate pyrochlore oxide nanoparticles with the composition (Gd_0.2_Nd_0.2_La_0.2_Pr_0.2_Sm_0.2_)_2_Sn_2_O_7_ through a simple glycine-assisted sol–gel synthesis. The phase evolution was probed at different heat-treatment temperatures from 1000 °C to 1500 °C. Among the temperatures investigated, a single-phase pyrochlore oxide was formed from 1300 °C without any impurity or phase separation. The obtained nanoparticles were characterized using various techniques, including X-ray diffraction (XRD), field-emission scanning electron microscopy (FESEM), nanoindentation, and dilatometry to investigate their physiochemical and mechanical properties. The Vickers hardness of high-entropy oxides is 4.2 ± 0.33 GPa, while a thermal expansion coefficient (TEC) of 8.7 × 10^−6^ K^−1^ at 900 °C is calculated. The results show that the prepared high-entropy pyrochlore oxide can be a suitable candidate for thermal barrier coating.

## 1. Introduction

Thermal barrier coatings are typically metal oxide layers that are usually coated over metallic parts to impede degradation of the base material under harsh operational environments, especially in gas turbines and jet engines [1]. During operation, the thermal barrier coating (TBC) materials must possess high melting points, low thermal conductivity, endurance to thermal cycling, and better sintering resistance with robust structural stability [2,3,4]. For example, yttrium-stabilized zirconium (YSZ) is considered a contemporary material in industries because it possesses relatively low thermal conductivity [3,5]. In addition, the point defects present in the oxide scatter heat-conducting phonons, effectively improving the resilience of the coating material [6]. However, phase transformation at high operating temperatures reduces the durability of YSZ thermal barrier coatings of YSZ [5,7].

There are a variety of metal oxide systems available as TBC materials, but the pyrochlore family is especially intriguing because of their compositional versatility and chemical inertness [8]. In addition, pyrochlore oxides possess high melting points along with a relatively high coefficient of thermal expansion and lower thermal conductivity [5]. This is due to the interplay between the individual principal elements, defect chemistry, and tunable properties. Therefore, utilizing pyrochlore oxides as a TBC material will be an excellent alternative to traditionally available stabilized or doped oxides [6,9,10,11,12].

In general, the pyrochlore structure is considered the superstructure of cubic fluorite when 1/8th of the oxygen atoms are removed in an ordered manner. Also, the lattice parameter of pyrochlore is twice that of a cubic fluorite lattice. Pyrochlore oxides are represented as A_2_B_2_O_7_ (where A and B are metal ions). Here, the A-site cations have a coordination number of eight, and the B-site cations have a coordination number of six. A and B sites can be designed by substituting pyrochlores for a variety of elements. As a result of structural tunability, pyrochlore oxides possess a plethora of unique physical and chemical properties and find applications in catalysis, photocatalysis, energy conversion, and storage devices [13,14,15,16].

Great efforts have been dedicated to creating new thermal barrier coatings apart from the existing materials. In recent years, high-entropy materials (HEMs) have gained a strong focus due to their unique elemental composition, resulting in better structural stability and functional properties [17,18,19]. As a result, the number of reported HEMs has increased notably. Especially, high-entropy oxides (HEOs), a subclass of high-entropy ceramics, have potential applications as a catalyst [20], photocatalyst [21,22,23], electrocatalyst [24,25], energy storage [26], electrochemical sensor [27], etc.

Utilizing HEOs as a TBC material will be interesting because of their structural complexity and stability. However, the real challenge lies in designing optimal TBC materials whose thermal expansion coefficient value must be similar to the base material to have better stability during operation, reducing thermal stress. Typically, the TEC of coating material must be in the range of 10–12 × 10^−6^ K^−1^ (typical of super alloys) to overcome thermal stress and delamination or cracking. To address this, HEO pyrochlores are an excellent choice tailored to match the performance of the base materials and can be tuned by optimizing the composition at the A and B sites.

To date, limited reports on stannate pyrochlore are available in the high-entropy field. Likewise, there are still many gaps for high-entropy rare earth stannates in the field of thermal barrier coating, which is less explored. Jiang et al. employed a chemical co-precipitation technique to synthesize (Y,Dy,Ce,Nd,La)_2_Sn_2_O_7_ stannate pyrochlore oxide as an anode material for lithium-ion batteries [28].

Trofimov et al. prepared ultra-high-entropy pyrochlore oxides (13RE)_2_A_2_O_7_ (A = Zr, Hf, Ti, Sn, Ce, or Pr) and investigated their phase evolution [29]. Apart from stannate pyrochlore, Zhang et al. prepared La_2_(Zr_0.2_Ti_0.2_Y_0.2_Yb_0.2_Nb_0.2_)_2_O_7_ pyrochlore oxide using a solid-state synthesis, and the thermal expansion coefficient value of 9.374 × 10^−6^ K^−1^ at 1000 °C was obtained [4]. Similarly, Vayer et al. prepared Dy_2_(Ti_0.2_Zr_0.2_Hf_0.2_Ge_0.2_Sn_0.2_)_2_O_7_ oxide by the ball milling technique and used a thermal expansion coefficient value of 10.3 × 10^−6^ K^−1^, whose values are close to nickel-based super alloys [30]. However, there seems to be scope for research related to designing new single-phase pyrochlore oxide compositions for TBC applications.

Therefore, this study aims to explore new compositions related to high-entropy stannate pyrochlore oxide employing a simple glycine-assisted sol–gel synthesis strategy. The list of recently reported high-entropy pyrochlore oxides, along with their investigated properties and applications, is tabulated in Table S1. Therefore, we find a wide opportunity to explore alternative TBC materials based on high-entropy stannate pyrochlore oxide.

In the present study, we synthesize a new composition (Gd_0.2_Nd_0.2_La_0.2_Pr_0.2_Sm_0.2_)_2_Sn_2_O_7_ using a glycine-assisted sol–gel technique, and its phase evolution was investigated. Here, the use of glycine serves as a chelating agent in stabilizing metal ions in the solution and preventing premature precipitation during the synthesis. In addition, it acts as a fuel during the gel-combustion step [31]. Elements involved in the present investigation were carefully selected due to their excellent thermal stability as pyrochlore oxides. In addition, dual oxidation states of praseodymium assist in stabilizing the pyrochlore structure without phase destabilization. Apart from the structural investigations, its mechanical properties and thermal expansion coefficient were measured. The results dictate that the formed high-entropy stannate pyrochlore oxide can be an alternative TBC material.

## 2. Materials and Methods

### 2.1. Materials and Reagents

Gadolinium(III) oxide (Gd_2_O_3_, 99.9%, Sigma-Aldrich, Moscow, Russia), neodymium(III) oxide (Nd_2_O_3_, 99+%, Sigma-Aldrich, Moscow, Russia), lanthanum(III) oxide (La_2_O_3_, 99.99%, Sigma-Aldrich, Moscow, Russia), praseodymium(III,IV) oxide (Pr_6_O_11_, 99.9%, Sigma-Aldrich, Moscow, Russia), samarium(III) oxide (Sm_2_O_3_, 99.9%, Sigma-Aldrich, Moscow, Russia), tin(II) chloride (anhydrous for synthesis, Sigma-Aldrich, Moscow, Russia), and glycine (NH_2_CH_2_COOH, ≥99.0% (NT), BioUltra, Sigma-Aldrich, Moscow, Russia) were all used as received without any further purification. Deionized (DI) water was used for the synthesis.

### 2.2. Synthesis of (Gd_0.2_Nd_0.2_La_0.2_Pr_0.2_Sm_0.2_)_2_Sn_2_O_7_ Oxide Nanoparticles

The synthesis of (Gd_0.2_Nd_0.2_La_0.2_Pr_0.2_Sm_0.2_)_2_Sn_2_O_7_ nanoparticles was carried out using a glycine-assisted sol–gel technique. The calculated amounts of respective oxides were weighed and added together into a beaker. For the current composition, the total concentration of all metal cations was 0.02 moles. Then, the required amount of HNO_3_ was added to the beaker, followed by stirring and heating the solution at 150 °C until all the oxides were dissolved. To this, the required amount of SnCl_2_ was added, followed by the addition of glycine (metal ions–glycine ratio was fixed at 1:1.4 moles). The stirring was continued for 15 min until homogenization was achieved and then stopped. While the heating was continued, a thick transparent gel was formed, followed by the evolution of gases like CO_2_, N_2_, H_2_O, and O_2_ [31]. Later, a spongy-textured foam was formed. Then, the heating was stopped, and the beaker was left to cool down to room temperature. The spongy foam was then powdered in an agate mortar and pestle, followed by calcination at different temperatures from 1000 °C to 1500 °C to investigate the phase evolution. The resultant powder was used for further physiochemical characterizations. The samples are denoted as HEO-0, HEO-1000, HEO-1100, HEO-1200, HEO-1300, HEO-1400, and HEO-1500, respectively, for as-synthesized, 1000 °C, 1100 °C, 1200 °C, 1300 °C, 1400 °C, and 1500 °C heat-treated samples.

### 2.3. Characterization

The phase evolution of heat-treated samples was investigated using a powder XRD diffractometer. The powdered samples were scanned from 20 to 80° with a scan speed of 5° per minute. Williamson–Hall (W-H) analysis was performed to assess the contribution of crystallite size and the lattice strain in XRD peak broadening. FESEM images were captured using a JEOL (JEOL JSM-7001F, JEOL, Tokyo, Japan) microscope operated at 20 kV. Sintering of the synthesized powder was carried out as follows. The as-synthesized powders were initially heat-treated at 500 °C for 2 h to remove unreacted precursors if present. Next, a pellet was prepared by compacting the powders on a die with a diameter of 10 mm by applying a load of 10 tons. The green pellet was then sintered at 1500 °C (RT-1000 °C (5 °C/min), 1000–1500 °C (2 °C/min)) for 2 hrs, followed by natural cooling. The pellet was then crushed in a mortar and pestle, followed by compaction and sintering again at 1500 °C for 5 h. Vickers hardness (Hv) was measured by using an FM-800 microhardness tester (Future-Tech Corp., Kawasaki, Japan). The coefficient of thermal expansion was measured using a dilatometer instrument (NETZSCH DIL 402 ExpedisClassic, NETZSCH, Selb, Germany) between 25 °C and 900 °C at a heating rate of 5 °C/min in air.

## 3. Results and Discussions

**Figure 1:**
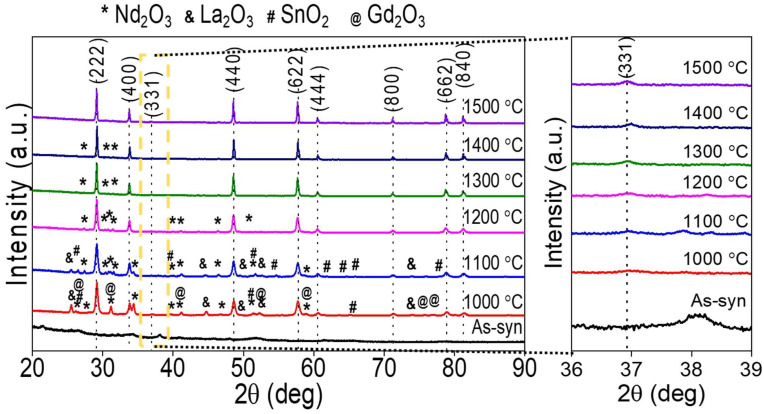
XRD patterns of as-synthesized high-entropy stannate pyrochlore oxide powders heat-treated at different temperatures (**left**). Enlarged XRD patterns from 36–39° displaying the evolution of superlattice reflection (331) at higher temperatures (**right**).

The XRD patterns of (Gd_0.2_Nd_0.2_La_0.2_Pr_0.2_Sm_0.2_)_2_Sn_2_O_7_ high-entropy stannate pyrochlore oxide are presented in Figure 1. The as-synthesized sample is completely amorphous in nature, as evidenced by broader reflections. When the calcination temperature was increased from 1000 °C to 1500 °C, crystalline phases evolved from amorphous precursors. At lower temperatures (1000 °C), peaks at 29.18°, 31.18°, 48.62°, 57.68°, 60.62°, 71.32°, 78.88°, and 81.44° correspond to the (222), (400), (331), (440), (622), (444), (800), (662), and (840) planes of a cubic pyrochlore (ICSD card number 01-087-1219, Pr_2_Sn_2_O_7_). Apart from these, other peaks are indexed to Nd_2_O_3_, La_2_O_3_, SnO_2_, and Gd_2_O_3_ systems, indicating phase separation or failure to form a single-phase solid solution. When the calcination temperature is increased from 1200 °C to 1500 °C, the intensity of Nd_2_O_3_, La_2_O_3_, SnO_2_, and Gd_2_O_3_ decreases, facilitating easy diffusion into the pyrochlore lattice, forming a single-phase solid solution. Especially at calcination temperatures starting from 1300 °C, a single-phase X-ray diffraction pattern is obtained and can be indexed to a pyrochlore oxide.

The ratio between the average ionic radius of A and B cations (r_A_/r_B_) is approximately 1.63. This value suggests that the designed composition falls under the pyrochlore family. However, a higher calcination temperature is required in order to achieve a single-phase solid solution. One possible reason is the Gibbs free energy of formation to achieve a single-phase solid solution. The Gibbs free energy of formation at constant temperature (T) and pressure (P) is given by ${△}_{formation}G={△}_{mixing}H-T\;{△}_{mixing}S$, where G is the Gibbs free energy, H is the enthalpy, and S is the entropy. In the case of pyrochlore oxides, the term $-T\;{△}_{mixing}S$ is large and negative only at high temperatures. Other possible factors that are involved in the phase formation at higher temperatures include solubility, ionic diffusion properties of individual metal cations. At lower temperatures, the diffusion is sluggish, while the energy required for the formation of metal oxides such as Nd_2_O_3_, La_2_O_3_, SnO_2_, and Gd_2_O_3_ is expected to be low compared to the energy required for pyrochlore oxide. Therefore, at lower temperatures, simpler oxides such as Pr_6_O_11_ and Sm_2_O_3_ easily diffuse into the pyrochlore lattice, while higher calcination temperatures are needed for the other oxides like Nd_2_O_3_, La_2_O_3_, SnO_2_, and Gd_2_O_3_.

Rietveld refinement was performed for the 1100–1500 °C samples, and the plots along with their corresponding values are shown in Figure S1 and Table S2. Except for the samples HEO-0, HEO-1000, and HEO-1100, the calculated X-ray diffraction fits well with the experimental XRD pattern. The estimated lattice parameter (10.5736 Ả for HEO-1500) is lower than the standard Pr_2_Sn_2_O_7_ (10.6004 Ả) stannous pyrochlore oxide. This can be explained by the incorporation of smaller cations such as Gd^3+^, Nd^3+^, and Sm^3+^ into the Pr^3+^ lattice, resulting in a reduced lattice parameter of high-entropy stannous pyrochlore oxide.

**Figure 2:**
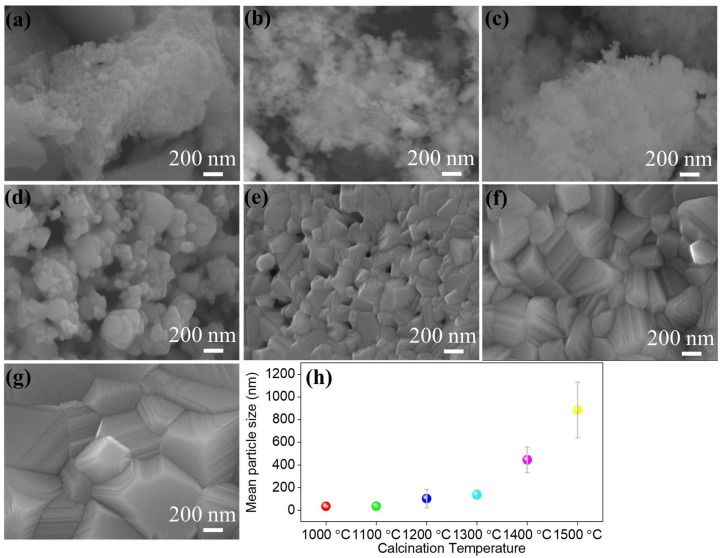
FESEM images of high-entropy stannate pyrochlore oxide nanoparticles calcined at different temperatures. ((**a**) As-syn, (**b**) 1000 °C, (**c**) 1100 °C, (**d**) 1200 °C, (**e**) 1300 °C, (**f**) 1400 °C, and (**g**) 1500 °C). (**h**) Calculated mean particle sizes for samples calcined at different temperatures.

To investigate the morphology of synthesized nanoparticles, FESEM was employed to observe the morphology, as shown in Figure 2. The as-synthesized powders contain strongly agglomerated spherical nanoparticles in the sub-nanometer range. With an increase in the calcination temperature, particle size starts to increase, which indicates that diffusion plays an important role. However, until 1100 °C, the growth of the nanoparticle is sluggish due to the presence of different-sized metal cations within the pyrochlore lattice.

Nevertheless, for the samples calcined at temperatures from 1200 °C to 1500 °C, we observe that the particle size tends to grow at a faster rate. Similarly, the particle shape changes from spherical into a fused cube-like morphology, indicating faster diffusion resulting in higher agglomerates. The particle size distribution plot (measured from 25 particles) is shown in Figure S2, and the mean particle size is shown in Figure 2h. The mean particle size increases from 36 ± 5 nm to 884 ± 245 nm. The sudden increase in the particle size at higher calcination temperatures can be correlated to the rate of diffusion of individual metal cations. In this case, the ionic radii of Nd^3^⁺ and La^3^⁺ are large compared to other metal cations. From the XRD results (Figure 1), it is evident that metal oxides like Nd_2_O_3_ and La_2_O_3_ remain as a secondary phase along with the pyrochlore phase when the calcination temperatures are less than 1200 °C. This reaction condition is unfavorable for the larger metal cations to diffuse into the pyrochlore lattice. However, a calcination temperature above 1300 °C provides an adequate thermal energy level for the diffusion of the constituent cations, facilitating the increased particle size observed from the FESEM results. Therefore, in the case of high-entropy systems, especially pyrochlore oxide, high-temperature heat treatment is essential.

**Figure 3:**
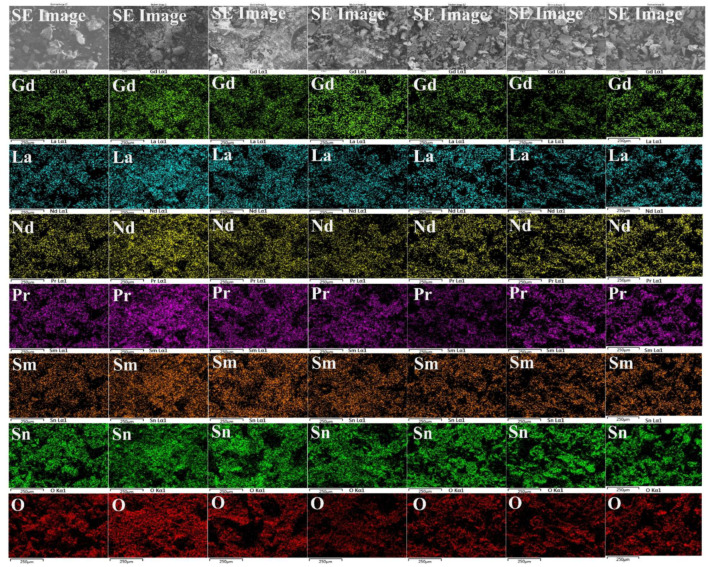
EDS mapping of high-entropy stannate pyrochlore oxide prepared at different calcination temperatures. (**Left** to **Right**: As-syn, 1000 °C, 1100 °C, 1200 °C, 1300 °C, 1400 °C, and 1500 °C). The scale bar represents 250 µm.

Elemental mapping was performed in order to evaluate the elemental distribution of high-entropy stannate pyrochlore oxide calcined at different temperatures (Figure 3). In all the samples, all the metal cations are evenly distributed within the nanoparticles. This indicates that no elemental segregation was evident. Similarly, the elemental composition computed from the EDS spectra (point scan) is tabulated in Table 1. In the case of as-synthesized samples, the elemental composition is not equimolar, which may be related to the formation of individual oxides, as confirmed from the XRD spectra. When the calcination temperature is increased, the rate of diffusion increases and an equimolar composition is achieved, forming a single-phase pyrochlore oxide.

**Figure 4:**
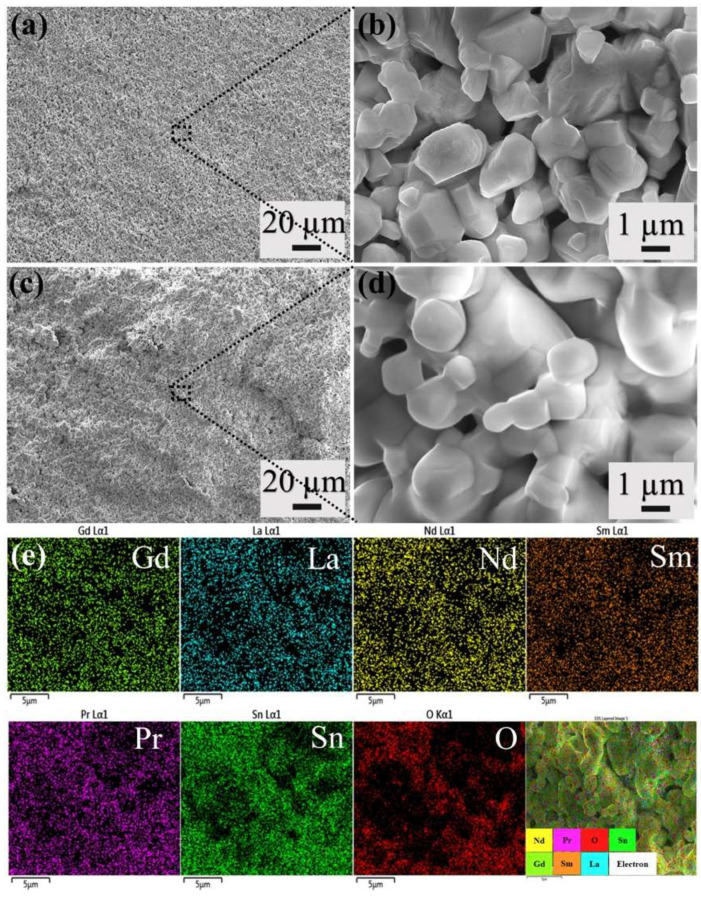
FESEM image of high-entropy stannate pyrochlore oxide (**a**,**b**) sintered pellet surface, (**c**,**d**) fractured surface, and (**e**) elemental mapping of fractured surface. **Left**: low magnification image and **right**: high magnification image. The scale bar in (**e**) represents 5 µm.

The prepared high-entropy stannous pyrochlore oxide powder was transformed into a pellet, and a double sintering step was performed at 1500 °C to achieve good density. One-step sintering at 1500 °C did not form a dense pellet, resulting in a brittle pellet that can be easily disintegrated. The surface of the sintered pellet is shown in Figure 4. The surface is even without any cracks but contains pores, which is evident from the high-magnification image (Figure 4b). The particles have sintered well, and the particle sizes are in the range of a few µm. Post-sintering, the sample contains pores, and this can be a result of diffusion occurring at high temperatures. During the process of sintering, smaller nanoparticles are consumed and transformed into micron-sized particles. As a result, the pores are trapped within the structure, which is common in pressure-less sintering [32]. Similarly, porous structures were observed for the fracture surface (Figure 4c,d), indicating homogeneous diffusion of particles occurring throughout the sample. In addition, the XRD result of the sintered oxide (Figure S3) confirms the existence of single-phase high-entropy stannate pyrochlore oxide and is supported by the EDS elemental mapping (Figure 4e).

The pores present in the sintered sample had an effect on the mechanical properties, such as hardness. Before measurements, the sintered sample was polished, the hardness was measured five times using a 500 g load with a dwell time of 10 s, and the mean hardness values were calculated. The calculated mean Vickers hardness of high-entropy stannate pyrochlore oxide is 4.2 ± 0.33 GPa, which is less compared to traditional pyrochlore oxides [33]. Smaller grain size is expected to improve the hardness of the material [34]. Because smaller grain size increases the number of grain boundaries, which in turn hamper local deformation, increasing the hardness values and suppressing crack propagation. However, the presence of pores in the high-entropy stannate pyrochlore oxide system weakens the overall mechanical property of the material [35]. Pores in the system act as stress concentrators and are more prone to deformation upon applied load [36]. In addition, the pores hinder grain boundary strengthening, reducing the overall mechanical properties. This can be improved by optimizing the sintering parameters and different sintering approaches, as well as the addition of sintering aids, which will be one of the future research directions [37,38].

**Figure 5:**
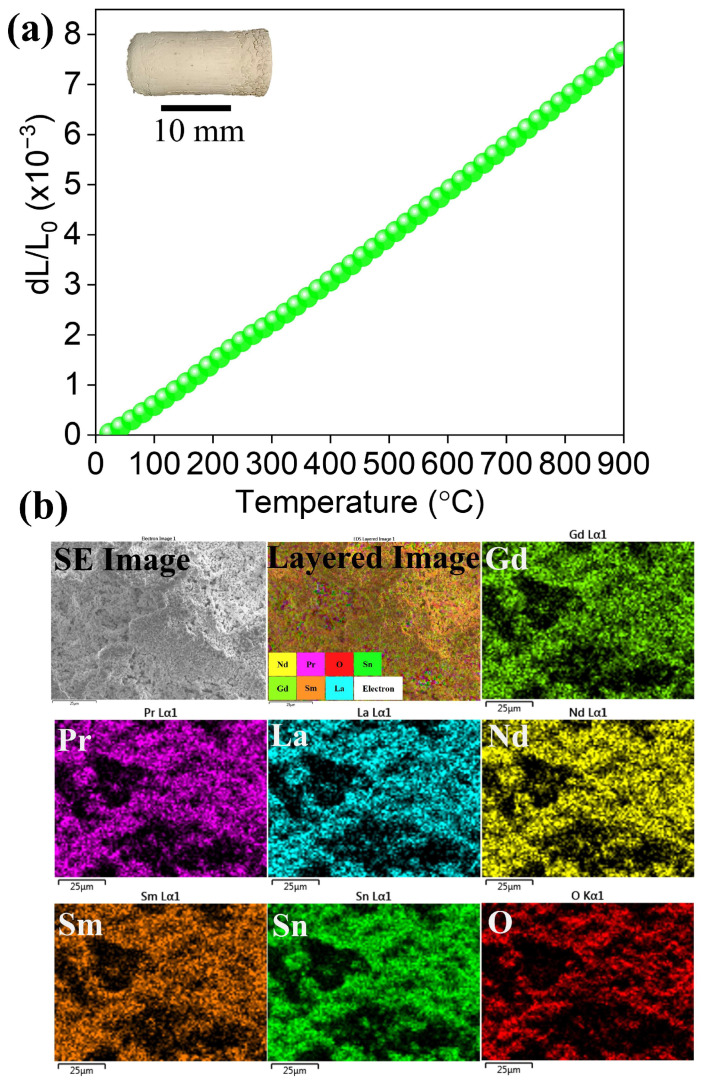
(**a**) The linear thermal expansion coefficients of synthesized high-entropy stannate pyrochlore oxide measured at various temperatures. Visual appearance of the sample after dilatometer study (inset) and (**b**) elemental mapping of the fractured sample after dilatometer investigation. The scale bar in (**b**) represents 25 µm.

For thermal barrier coating applications, it is crucial to have a thermal expansion coefficient in line with that of the base materials. As a result, the performance of turbine blades will be improved, preventing cracks and lowering the thermal stress at the coating interface. Therefore, the thermal expansion coefficient is a crucial parameter that decides the performance of the coating materials. The linear thermal expansion coefficient with respect to different temperatures is shown in Figure 5a. With an increase in temperature, the change in length is linear, which is attributed to lattice spacing expansion [39]. The high-entropy stannate pyrochlore oxide is structurally stable during the measurement temperatures without any abrupt volume changes evident from the linear thermal expansion. The thermal expansion coefficient of sintered high-entropy stannate pyrochlore oxide was calculated using Equation (1),

(1) αT1−T2 =(△LL0)(T2)−(△LL0)(T1)T2−T1

where *α* is the average change of length for a unit length sample between the range of temperatures *T*_1_ and *T*_2_, and (ΔL/L_0_) is the average change of length for unit length. Accordingly, the estimated value of 8.7 × 10^−6^K^−1^ at 900 °C is obtained, which is lower than that of yttria-stabilized zirconia [40]. The TEC values are in line with Al_2_O_3,_ suggesting that the prepared high-entropy stannate pyrochlore oxide will be a promising candidate as a thermal barrier coating. Table 2 lists the reported TEC of various high-entropy oxide systems that possess lower TEC. This will assist in better thermal stability of the coating by reducing the thermal mismatch of the coating material and the base material, which is advantageous. Similarly, under extreme stress and thermal cycling processes, lower TEC is expected to reduce the thermal cracking of the coating material. The elemental mapping of high-entropy stannate pyrochlore oxide after the dilatometer studies indicates that the elemental distribution is unaffected, proving the structural stability of high-entropy stannate pyrochlore oxide (Figure 5b), supported by a single-phase XRD pattern (Figure S4). Further research is necessary to explore its complete functional applications as a thermal barrier coating material, which will be investigated in our future work.

**Table 2:** Reported thermal expansion coefficient values of various oxides in comparison with our synthesized high-entropy stannate pyrochlore oxide.

S.No.	Composition	Thermal Expansion Coefficient ×10^−6^ K^−1^	Reference
1	(Dy_0.2_Nd_0.2_Sm_0.2_Eu_0.2_Yb_0.2_)_2_Zr_2_O_7_	10.59 (1500 °C)	[12]
2	(La_0.2_Gd_0.2_Y_0.2_Sm_0.2_Ce_0.2_)_2_Zr_2_O_7_	11.1 (1000 °C)	[41]
3	(La_0.2_Nd_0.2_Sm_0.2_Eu_0.2_Gd_0.2_)_2_Ce_2_O_7_	12 (1400 °C)	[11]
4	(La_0.2_Y_0.2_Sm_0.2_Eu_0.2_Gd_0.2_)_2_Zr_2_O_7_	11 (1200 °C)	[42]
5	(Y_0.3_Gd_0.3_Yb_0.4_)_4_Hf_3_O_12_	11 (1500 °C)	[43]
6	(La_0.3_Gd_0.3_Ca_0.4_)_2_(Ti_0.2_Zr_0.2_Hf_0.2_Nb_0.2_Ta_0.2_)_2_O_7_	9.0 (1200 °C)	[10]
7	Dy_2_(Ti_0.2_Zr_0.2_Hf_0.2_Ge_0.2_Sn_0.2_)_2_O_7_	10.3	[30]
8	(La_0.2_Sm_0.2_Er_0.2_Yb _0.2_Y_0.2_)_2_Ce_x_O_3+2x_ (x = 4.4)	13.12 (850 °C)	[39]
9	La_2_(Zr_0.2_Ce_0.2_Hf_0.2_Sn_0.2_Ti_0.2_)_2_O_7_	9.67 (1000 °C)	[44]
10	Er_2_(Y_0.2_Yb_0.2_Nb_0.2_Ta_0.2_Ce_0.2_)_2_O_7_	10.56	[45]
11	La_2_(Zr_0.2_Ti_0.2_Y_0.2_YB_0.2_Nb_0.2_)_2_O_7_	9.374 (1000 °C)	[4]
12	(Gd_0.2_Nd_0.2_La_0.2_Pr_0.2_Sm_0.2_)_2_Sn_2_O_7_	8.702 (900 °C)	This work

## 4. Conclusions

We have successfully prepared a new high-entropy stannate pyrochlore as a thermal barrier coating material with a composition of (Gd_0.2_Nd_0.2_La_0.2_Pr_0.2_Sm_0.2_)_2_Sn_2_O_7_ using a simple glycine-assisted sol–gel synthesis. The phase evolution and phase stability were evaluated at various temperatures from 1000 °C to 1500 °C. A single-phase stannate pyrochlore oxide was obtained when the samples were heat-treated at 1300 °C. The FESEM images show that the size of the nanoparticle abruptly increased from 36 nm to 884 nm, indicating stronger diffusion of metal cations, which are responsible for the formation of a single phase. The presence of pores during the sintering stage impacts the Vickers hardness (4.2 GPa), which necessitates the need for optimization. However, the calculated TEC values are in line with the TEC of the base material, which can be a promising candidate for thermal barrier coating application.

## Figures and Tables

**Table 1:** Elemental composition of high-entropy stannate pyrochlore oxide computed from the EDS spectra.

Calcination Temperature (°C)	Element (at %)					
	Sn	La	Pr	Nd	Sm	Gd
As-synthesized	38.92	13.81	11.68	12.47	11.68	11.45
1000	41.44	12.90	11.93	11.06	11.26	11.41
1100	43.37	11.29	11.31	11.12	11.56	11.35
1200	47.72	11.34	9.92	9.18	11.01	10.82
1300	48.60	11.98	9.77	8.86	10.13	10.65
1400	49.59	10.30	10.02	9.12	10.35	10.62
1500	50.18	10.97	9.38	9.83	9.64	9.99

## Data Availability

Data are available on request from the corresponding author.

## Supplementary Materials

The following supporting information can be downloaded at: https://www.mdpi.com/article/10.3390/nano15120939/s1, Table S1: Recently reported high-entropy pyrochlore oxide systems; Figure S1: Rietveld refinement fit of high-entropy stannate pyrochlore (Gd_0.2_Nd_0.2_La_0.2_Pr_0.2_Sm_0.2_)_2_Sn_2_O_7_ oxide calcined at different temperatures ((a) 1100 °C, (b) 1200 °C, (c) 1300 °C, (d) 1400 °C, and (e) 1500 °C).; Table S2. Summary of refined values of high-entropy stannate pyrochlore oxide powder obtained from the FullProf software.; Figure S2: Particle size distribution of high-entropy stannate pyrochlore oxide estimated from the SEM images. (a) 1000 °C, (b) 1100 °C, (c) 1200 °C, (d) 1300 °C, (e) 1400 °C, and (f) 1500 °C.; Figure S3: (a) XRD pattern of sintered pellet prepared from high-entropy stannate pyrochlore oxide powder.; Figure S4: XRD pattern of high-entropy stannate pyrochlore oxide sample after dilatometer study. References [28,46,47,48,49,50,51,52,53,54,55,56] are included in the Supplementary Materials.

## Abbreviations

The following abbreviations are used in this manuscript:

EDS	Energy Dispersive X-ray Spectroscopy
FESEM	Field-emission scanning electron microscopy
HEM	High-entropy materials
HEO	High-entropy oxide
TBC	Thermal barrier coating
W-H	Williamson–Hall
XRD	X-ray diffraction
YSZ	Yttrium-stabilized zirconium
