# Supplementary material for: Phase Evolution of High-Entropy Stannate Pyrochlore Oxide Synthesized via Glycine-Assisted Sol–Gel Synthesis as a Thermal Barrier Coating Material
Source: Nanomaterials (Basel). 2025 Jun 17;15(12):939. doi: 10.3390/nano15120939 (PMC12195986; doi:10.3390/nano15120939)
Supplement: Supplementary file 1 [file nanomaterials-15-00939-s001.zip › nanomaterials-3675618-supplementary.pdf]

Supporting Information for

Phase Evolution of High-Entropy Stannate Pyrochlore Oxide Synthesized via Glycine-Assisted Sol–Gel Synthesis as a Thermal Barrier Coating Material

Mariappan Anandkumar [1,*], Kannan Pidugu Kesavan [2], Shanmugavel Sudarsan [3,4], Dmitry Evgenievich Zhivulin [5], Natalia Aleksandrovna Shaburova [6], Ahmad Ostovari Moghaddam [7], Ksenia Sergeevna Litvinyuk [1] and Evgeny Alekseevich Trofimov [6,*]

[1] High-Entropy Materials Research Laboratory, South Ural State University, Chelyabinsk 454080, Russia; litviniukks@susu.ru
[2] Department of Physics, PSG Institute of Technology and Applied Research, Coimbatore 641 062, India; kannan@psgitech.ac.in
[3] Department of Chemistry, Saveetha Engineering College, Chennai 602 105, India; srsudarsan29@gmail.com
[4] Laboratory of Problems of Recycling Modern Multicomponent Materials with Complex Structure, South Ural State University, Chelyabinsk 454080, Russia
[5] Regional Youth Laboratory of Electromechanical, Electronic and Electrochemical Systems, South Ural State University, Chelyabinsk 454080, Russia; zhivulinde@susu.ru
[6] Department of Materials Science, Physical and Chemical Properties of Materials, South Ural State University, Chelyabinsk 454080, Russia; shaburovana@susu.ru
[7] Department of Applied Mathematics, National Research University Higher School of Economics, Moscow 101000, Russia; mostovari@hse.ru
* Correspondence: drmaksmile@gmail.com or anandkumarmariappan@susu.ru (M.A.); trofimovea@susu.ru (E.A.T.); Tel.: +91-7418616336 (M.A.)

Table S1: Recently reported high-entropy pyrochlore oxide systems

S.No	Composition	Synthesis method	Properties/Application	Ref
1	$(Yb_{0.2}Tm_{0.2}Lu_{0.2}Ho_{0.2}Er_{0.2})_2Ti_2O_7$	Floating-zone growth technique	Irradiation-induced amorphization	[1]
2	$(Nd_2(Ti_{0.25}Zr_{0.25}Hf_{0.25}Sn_{0.25})_2O_7)$ and $(Nd_2(Zr_{0.3}Hf_{0.2}Sn_{0.2}Nb_{0.2}Ce_{0.1})_2O_7)$	Solid state	Immobilizing radioactive nuclides in molten salt radwastes	[2]
3	(La1/8Sm1/8Nd1/8Pr1/8Y1/8Gd1/8Dy1/8Yb1/8)2(Hf1/2Zr1/2)2O7	Glycine nitrate procedure (GNP)	Structural stability and high-pressure behavior	[3]
4	$(Y_{0.2}Ho_{0.2}Dy_{0.2}Gd_{0.2}Pr_{0.2})_2Ru_2O_7$	-	Electrocatalyst for acid-water oxidation	[4]
5	$(La_{1/7}Nd_{1/7}Sm_{1/7}Eu_{1/7}Gd_{1/7}Dy_{1/7}Ho_{1/7})_2Zr_2O_7$	Reactive spark plasma sintering	Mechanical	[5]
6	$(La_{0.2}Nd_{0.2}Sm_{0.2}Gd_{0.2}Yb_{0.2})_2(Zr_{0.75}Ce_{0.25})_2O_7$	Solid-state sintering	Mechanical	[6]
7	$Gd_2(Ti_{0.2}Zr_{0.2}Sn_{0.2}Hf_{0.2}Ta_{0.2})_2O_7$ and $Gd_2(Ti_{0.2}Zr_{0.2}Sn_{0.2}Hf_{0.2}Nb_{0.2})_2O_7$	Solid-state	Phase transformation upon Kr^{2+} irradiation	[7]
8	$(Y,Dy,Ce,Nd,La)_2Sn_2O_7$	Co-precipitation	Anode material for lithium-ion batteries	[8]

9	$(Ho_{0.2}Y_{0.2}Dy_{0.2}Gd_{0.2}Eu_{0.2})_2Ti_2O_7/TiO_2$	Rapid melt solidification	Mechanical and thermal	[9]
10	$(La_{0.2}Nd_{0.2}Sm_{0.2}Eu_{0.2}Gd_{0.2})_2Zr_2O_7$	Reactive flash sintering	Phase evolution and densification	[10]
11	$(RE_{0.2}Gd_{0.2}Ho_{0.2}Er_{0.2}Yb_{0.2})_2Ti_2O_7$ (RE = Sm, Y, Lu)	Solid-phase reaction	-	[11]
12	$(La_{0.2}Nd_{0.2}Sm_{0.2}Eu_{0.2}Gd_{0.2})_2Zr_2O_7$	Solid-state	Mechanical, thermal	[12]

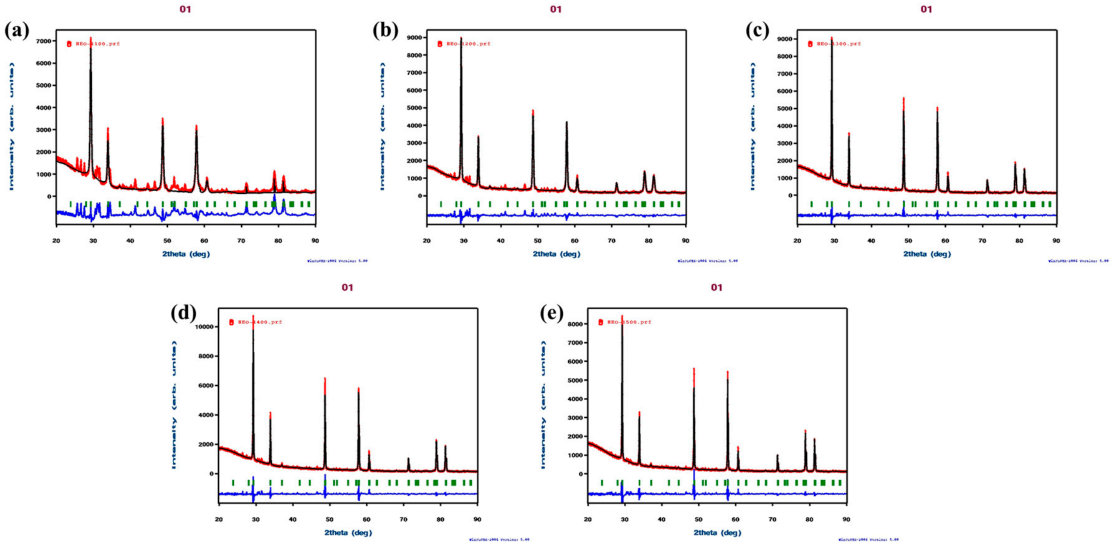

Figure S1: Rietveld refinement fit of high-entropy stannate pyrochlore $(Gd_{0.2}Nd_{0.2}La_{0.2}Pr_{0.2}Sm_{0.2})_2Sn_2O_7$ oxide calcined at different temperatures ((a) 1100 °C, (b) 1200 °C, (c) 1300 °C, (d) 1400 °C, and (e) 1500 °C).

Table S2. Summary of refined values of high-entropy stannate pyrochlore oxide powder obtained from the FullProf software.

**Sample** $(Gd_{0.2}Nd_{0.2}La_{0.2}Pr_{0.2}Sm_{0.2})O_2$	**Lattice parameter (Å)**	$\mathbf{R_p}$ **(%)**	$\mathbf{R_{wp}}$ **(%)**	$\mathbf{R_{exp}}$ **(%)**	χ^2	**Goodness of Fit**
**As-Syn**	-	-	-	-	-	-
**1000 °C**	-	-	-	-	-	-
**1100 °C**	10.5725	38.7	38.6	8.01	23.26	4.81
**1200 °C**	10.5790	23.8	18.9	9.77	3.76	1.93
**1300 °C**	10.5748	21	15.6	10.2	2.35	1.52
**1400 °C**	10.5766	24.1	18.4	9.8	3.51	1.87
**1500 °C**	10.5736	27.4	21.4	10.8	3.96	1.98

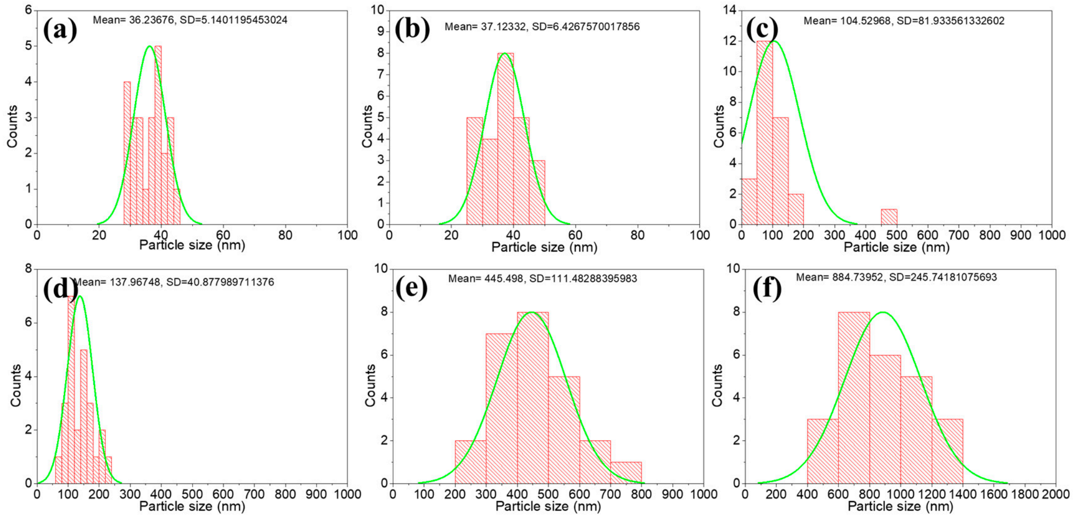

Figure S2: Particle size distribution of high-entropy stannate pyrochlore oxide estimated from the SEM images. (a) 1000 °C, (b) 1100 °C, (c) 1200 °C, (d) 1300 °C, (e) 1400 °C, and (f) 1500 °C.

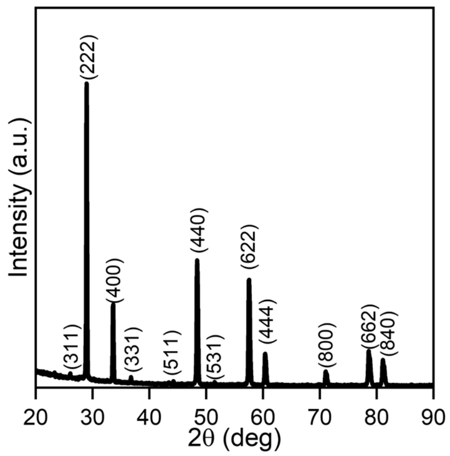

Figure S3: (a) XRD pattern of sintered pellet prepared from high-entropy stannate pyrochlore oxide powder.

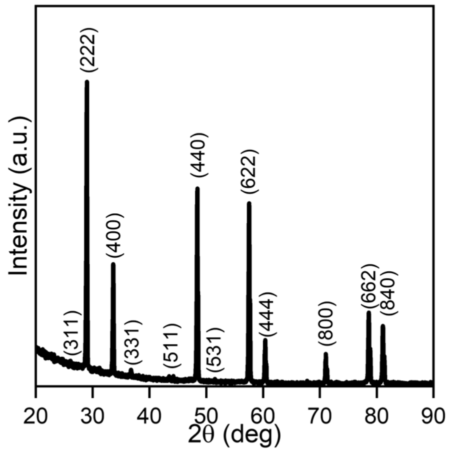

Figure S4: XRD pattern of high-entropy stannate pyrochlore oxide sample after dilatometer study.

References:

[1] W.J. Weber, C. Kinsler-Fedon, V. Keppens, Y. Zhang, A.H. Mir, Temperature dependence of irradiation-induced amorphization in a high-entropy titanate pyrochlore, MRS Communications, 14 (2024) 1364-1370.

[2] K. Zhang, X. Duan, M. Jiang, X. Liu, Z. Qian, Q. Zhang, Y. Qiao, Design and synthesis of high-entropy $A_2B_2O_7$-type pyrochlore ceramics for the immobilization of molten salt radwastes, Ceramics International, 50 (2024) 52640-52648.

[3] B. Matović, N.M. Belozerova, D.P. Kozlenko, I.Y. Zel, J. Maletaškić, D. Zagorac, S. Butulija, I. Cvijović-Alagić, High-pressure behavior of high-entropy $A_2B_2O_7$ pyrochlore, Ceramics International, 50 (2024) 52649-52654.

[4] J. Zhang, L. Shi, X. Miao, L. Yang, S. Zhou, A new-type high-entropy electrocatalyst with a pyrochlore structure for acid-water oxidation, Journal of Materials Chemistry A, 12 (2024) 12785-12794.

[5] Z. Teng, P. Wang, S. Zeng, W. Feng, C. Chen, P. Jia, Y. Tan, S. Peng, Reactive spark plasma sintering of high-entropy $(La_{1/7}Nd_{1/7}Sm_{1/7}Eu_{1/7}Gd_{1/7}Dy_{1/7}Ho_{1/7})_2Zr_2O_7$ pyrochlore ceramic, Ceramics International, 50 (2024) 6892-6897.

[6] D. Guo, F. Zhou, B. Xu, Y. Wang, Y. Wang, High-entropy $(La_{0.2}Nd_{0.2}Sm_{0.2}Gd_{0.2}Yb_{0.2})_2(Zr_{0.75}Ce_{0.25})_2O_7$ thermal barrier coating material with significantly enhanced fracture toughness, Chinese Journal of Aeronautics, 36 (2023) 556-564.

[7] Y. Li, Y. Lei, S. Zhao, H. Xiao, H. Liu, Y. Wang, Y. Luo, J. Zhang, J. Wang, R.C. Ewing, C. Wang, Phase transformation and radiation resistance of B-site high entropy pyrochlores, Scripta Materialia, 229 (2023) 115367.

[8] T. Jiang, F. Wu, Y. Ren, J. Qiu, Z. Chen, Pyrochlore phase $(Y,Dy,Ce,Nd,La)_2Sn_2O_7$ as a superb anode material for lithium-ion batteries, Journal of Solid State Electrochemistry, 27 (2023) 763-772.

[9] Y. Guo, R. Zheng, S. Feng, J. Fu, Y. Yang, H. Wang, Z. Hao, J. Li, High-entropy $(Ho_{0.2}Y_{0.2}Dy_{0.2}Gd_{0.2}Eu_{0.2})_2Ti_2O_7/TiO_2$ composites with excellent mechanical and thermal properties, Journal of the European Ceramic Society, 43 (2023) 6398-6406.

[10] G. Zhao, S. Cai, Y. Zhang, H. Gu, C. Xu, Reactive flash sintering of high-entropy oxide $(La_{0.2}Nd_{0.2}Sm_{0.2}Eu_{0.2}Gd_{0.2})_2Zr_2O_7$: Microstructural evolution and aqueous durability, Journal of the European Ceramic Society, 43 (2023) 2593-2600.

[11] S. Zhu, J. Zhu, S. Ye, K. Yang, M. Li, H. Wang, J. He, High-entropy rare earth titanates with low thermal conductivity designed by lattice distortion, Journal of the American Ceramic Society, 106 (2023) 6279-6291.

[12] J. Zhu, X. Meng, P. Zhang, Z. Li, J. Xu, M.J. Reece, F. Gao, Dual-phase rare-earth-zirconate high-entropy ceramics with glass-like thermal conductivity, Journal of the European Ceramic Society, 41 (2021) 2861-2869.
